# Interleukin-1/-33 Signaling Pathways as Therapeutic Targets for Endometriosis

**DOI:** 10.3389/fimmu.2019.02021

**Published:** 2019-08-22

**Authors:** Toru Kato, Koubun Yasuda, Kazufumi Matsushita, Ken J. Ishii, Seiichi Hirota, Tomohiro Yoshimoto, Hiroaki Shibahara

**Affiliations:** ^1^Department of Obstetrics and Gynecology, Hyogo College of Medicine, Nishinomiya, Japan; ^2^Department of Immunology, Hyogo College of Medicine, Nishinomiya, Japan; ^3^Division of Vaccine Science, Department of Microbiology and Immunology, The Institute of Medical Science, University of Tokyo, Tokyo, Japan; ^4^Department of Surgical Pathology, Hyogo College of Medicine, Nishinomiya, Japan

**Keywords:** MyD88, IRAK4, ST2, IL-1R1, estrogen

## Abstract

Endometriosis is an estrogen-dependent disease with symptoms of dysmenorrhea, chronic pain, and infertility that affects 6–10% of women of reproductive age. Medical or surgical therapy, such as administration of an anti-gonadotropin or ovarian cystectomy, provide effective pain relief. However, neither therapy can be used for patients wishing to become pregnant. Despite the high morbidity, the pathogenesis of endometriosis has not been well-elucidated. Several inflammatory cytokines are reported to participate in the onset of endometriosis. Here, we examined the role of interleukin (IL)-1/IL-33 signaling in the development of endometriosis using a mouse model of endometriosis. Endometriotic lesion volume was significantly reduced in *Il33*^−/−^ and *Il1r1*^−/−^ mice, and almost completely suppressed in *Myd88*^−/−^ mice. Mice intraperitoneally administered with an antibody against IL-1 receptor 1 (IL-1R1) or IL-33 developed limited endometriotic lesions. Oral administration of an inhibitor against IL-1R-associated kinase 4 (IRAK4), a downstream signal molecule of MyD88, also suppressed lesion formation. Furthermore, even after the development of cystic lesions the IRAK4 inhibitor prevented the enlargement of lesions. These treatments all significantly reduced cellular proliferation, shown by decreased Ki-67 expression. These results reveal that IL-1/IL-1R1, IL-33/IL-33R and associated downstream signaling molecules are involved in the pathogenesis of endometriosis, and may provide novel therapeutic targets for endometriosis.

## Introduction

Endometriosis is defined as endometrial tissue that occurs outside the uterine cavity, and an estrogen-dependent disease with predominant symptoms of dysmenorrhea, dyspareunia, chronic pain, and infertility. Endometriosis affects 6–10% of women of reproductive age (1) and up to 50% of women with infertility (2), highlighting its negative impact upon female reproductive health. It is also associated with a moderate increase in ovarian cancer risk (3). The pathogenesis of endometriosis appears to be multifactorial, including ectopic endometrial tissue, altered immunity, imbalanced cell proliferation and apoptosis, aberrant endocrine signaling, and genetic factors. The endometrial transplantation theory by Sampson is one possible cause of endometriosis, involving reflux of menstrual blood into the abdominal cavity via the oviduct (4). However, because the reflux of menstrual blood is observed in most women, endometriosis cannot be explained by reflux alone. In addition, inflammatory cytokines, such as tumor necrosis factor α (5, 6), interleukin (IL)-1β (7, 8), and IL-33 (9), and immune cells, such as macrophages (10) and natural killer cells (11, 12), were reported to be involved in the pathogenesis of endometriosis. However, limited studies have investigated the mechanism of their actions in endometriosis.

IL-33 is a member of the IL-1 family cytokines that stimulates various cell types, such as Th2 cells, mast cells and group 2 innate lymphoid cells (ILC2s), and it has pleiotropic functions (13–15). Various types of cells such as epithelial cells and endothelial cells express IL-33 in the nucleus and release it in response to cellular stress (16, 17). In general, IL-1 family member cytokines, including IL-1β and IL-18, are synthesized as precursor proteins in the cytoplasm, and become active after processing in the inflammasome. In contrast, the full-length form of IL-33 is active, and processing by proteases enhances its activity, whereas cleavage by caspases abrogates its function (18). IL-33 is important for innate-type mucosal immunity in the lungs and gut, and airway inflammation, as well as peripheral antigen-specific responses by induction of Th2 cytokines (19–22).

Interleukin-1 is the most important proinflammatory cytokine and is mainly produced by monocytes/macrophages (23). Both IL-1α and IL-1β use a heterodimeric receptor complex consists of IL-1 receptor 1 (IL-1R1) and IL-1R accessory protein (IL-1RAcP) for the induction of inflammatory processes (24). The IL-1R uses the signal transduction adaptor protein MyD88, which recruits a serine/threonine kinase IL-1R-associated kinase 4 (IRAK4), resulting activation of transcription factor NF-κB and mitogen-activated protein kinases (25, 26). IL-33 uses a receptor complex of ST2 (IL-1R-like 1) and IL-1RAcP for its receptor, and also uses MyD88/IRAK4 signaling pathway. ST2 has two forms, a full-length membrane-bound form and a short secreted form (soluble ST2) consisting of an extracellular domain. The membrane-bound form acts as a receptor for IL-33 and the secreted form neutralizes IL-33 as a decoy receptor.

Currently, almost all drugs for the treatment of endometriosis are suppressive, not curative. Medical therapy with non-steroidal anti-inflammatory drugs (NSAIDs), oral contraceptives or anti-gonadotropin provides effective pain relief (27), but does not resolve the endometriosis. Surgical therapy is more effective for pain reduction and endometriosis-associated symptoms (28). However, after medical or surgical treatment, there is a high recurrence frequency of endometriosis; 21.5% at 2 years and 40–50% at 5 years (29). Furthermore, either therapy is limited to patients who do not wish to become pregnant (30). The limitations of currently available options prompted us to seek novel therapies for endometriosis. Here, we used an experimental mouse model to determine the molecular mechanisms underlying endometriosis and examine novel drug targets for endometriosis. We investigated the role of IL-33, IL-1R1, and MyD88 in the development of endometriosis in mice, and demonstrated the potential of inhibitors for IL-1/IL-33 and their signaling pathway as therapeutic targets for endometriosis.

## Materials and Methods

### Ethics Statement

This study was carried out in accordance with recommendations of the Regulations for Animal Experimentation in Hyogo College of Medicine, Hyogo College of Medicine Animal Experiment Committee. The protocols were approved by the Hyogo College of Medicine Animal Experiment Committee (No. 14-028, 16-087).

### Mice

Wild type (WT) BALB/c and C57BL/6 mice were purchased from Charles River Laboratories Japan (Yokohama, Japan). BALB/c-background *Tlr4*^−/−^ mice were obtained from Oriental BioService Inc. (Kyoto, Japan). C57BL/6-background *Il1r1*^−/−^ mice were purchased from The Jackson Laboratory. BALB/c-background *Myd88*^−/−^, *Il18*^−/−^, *Il33*^−/−^, and *Il1rl1*^−/−^ mice were generated as described (20, 25, 31). Mice were maintained under specific pathogen-free conditions at the animal facilities of the Hyogo College of Medicine.

### Mouse ST2-Human Fc Fusion Protein (mST2Fc)

The mST2Fc was made by Mitsubishi Tanabe Pharma. The sequence of mouse ST2 ectodomain (amino acids 1–332) fused to the N-terminus of the Fc region of human IgG1 was inserted into the mammalian expression vector pEE12.4 (Lonza Biologics, Slough, UK). The expressed mST2Fc was purified by Protein A chromatography, followed by hydroxyapatite chromatography. The fusion protein was concentrated and buffer-exchanged to PBS (pH 7.4). Protein concentration was determined by the Pierce BCA Protein Assay Kit (Thermo Scientific, Rockford, IL, USA).

### Mouse Endometriosis Model

The experimental endometriosis model was made as described previously with minor modification (32, 33). Six-week-old female mice were ovariectomized under anesthesia with isoflurane, and treated with estradiol subcutaneously (0.5 μg/week/mouse, Fuji Pharma, Tokyo, Japan). Two weeks later, mice were randomly divided into two groups, donor and recipient. Donor group mice were euthanized by cervical dislocation, then uteri were removed, minced, and suspended in PBS containing ampicillin (1 mg/ml). Uteri samples were transplanted (40 mg/recipient) into the peritoneal cavity of recipient mice under isoflurane-induced anesthesia, using a 1 ml syringe (without a needle) into a small median abdominal incision. The control group received 400 μl of ampicillin/PBS. Unless otherwise indicated, the donor and recipient mice were of the same strain. Two weeks after transplantation, mice were euthanized by cervical dislocation and lesion size in the abdominal cavity was evaluated (Supplementary Figure 1). There are several lesions with various sizes in each mouse. The lesion volume was calculated using the following formula: V = 4/3πA^2^a; A: long radius, a; short radius. The sum of each lesion volume was taken as the total volume. Lesion sections were photographed with an iPhone (scale: mm). There are several lesions with different appearances (white, red or brown). The reason for these differences is unknown.

In some experiments, mice were administered 100 ng recombinant human IL-33 (rhIL-33, HOKUDO, Sapporo, Japan) (20) intraperitoneally (ip) on day 1, 3, 5, 8, 10, and 12 after transplantation of uterine samples.

For neutralization of human IL-33, mice were administered anti-human IL-33 antibody (10 mg/kg ip, A10-1C04, provided from Mitsubishi Tanabe Pharma, Yokohama, Japan, Patent No. WO2015099175) once a week. Normal human IgG1 (Eureka therapeutics, CA) was used as a control. For neutralization of murine IL-33, mice were administered mST2Fc (20 mg/kg iv) once in 3 days. Human IgG1-Fc (Bio X cell, NH) was used as a control. For IL-1 neutralization, mice were administered (iv) 250, 125, and 125 μg anti-IL-1R1 antibody (35F5, #553693, BD Biosciences, CA) at day −1, day 4, and day 8, respectively. Anti-horseradish peroxidase rat IgG1 (HRPN, BP0088, BioXcell) was used as a control.

For blocking IRAK4 signaling, an IRAK4 inhibitor (AS2444697; Sigma-Aldrich Japan, Tokyo, Japan) was solubilized at 10 mg/ml with DMSO and diluted with water, then orally administered twice a day at 5 mg/kg for 5 days after transplantation, or 10 mg/kg for 14 days from 2 weeks after transplantation.

### Flow Cytometry

The uteruses from untreated WT and ST2 deficient mice (*Il1rl1*^−/−^) were minced and digested with complete medium (RPMI 1640 supplemented with 10% fetal bovine serum, 50 μM 2-ME, 2 mM L-glutamine, 100 U/ml penicillin, and 100 μg/ml streptomycin) containing 400 U/ml collagenase (032-22364, Wako, Osaka, Japan) and 10 μg/ml DNase I (11284932001, Roche, Mannheim, Germany) for 60 min at 37°C. Cell suspensions were filtered using a cell strainer. Uterus cells were stained with antibodies for CD45 (I3/2.3, PerCP-Cy5.5, 147706, BioLegend, CA), EpCAM1 (G8.8, APC, 118213, BioLegend), ST2 (DJ8, Biotin, 101001B, MD BioSciences, MN), and MAdCAM1 (MECA-367, AlexaFluor 488, 120708, BioLegend) followed by streptavidin (Brilliantviolet 421, 405225, BioLegend). Data were acquired using SP6800 flow cytometer (SONY, Tokyo, Japan) and analyzed using FlowJo software (Tree Star Inc., OR).

### Histological Analysis

For histological analysis, lesion specimens were fixed with 4% (w/v) paraformaldehyde (PFA) and embedded in paraffin. Specimens were sectioned at 5-μm thickness and deparaffinized sections were stained with hematoxylin and eosin.

For immunohistochemical staining, deparaffinized sections were microwave-heated in citrate buffer (pH 6.0) for antigen retrieval, then blocked with 1% BSA, stained with rabbit anti-mouse Ki-67 polyclonal antibody (ab16667, Abcam, Tokyo, Japan) or anti-mouse ERβ (Santa Cruz, TX) and detected by Envision+ Dual link (K4063, DAKO, CA). After development with 3-amino-9-ethylcarbazole, specimens were stained with hematoxylin. Sections were evaluated by light microscopy (AX80, Olympus Lifescience, Tokyo, Japan). For analysis of proliferating cells, sections were observed by microscopy to calculate Ki-67 positive nuclei in epithelial cells. The Ki-67 positive epithelial cells were counted in three fields of view per slide and averaged. To detect apoptotic cells, sections of cystic lesions were stained using TUNEL with an Apoptosis *in situ* Detection Kit (FUJIFILM Wako Pure Chemical, Osaka, Japan), as per manufacturer's instruction. For analysis of apoptotic cells, sections were observed by light microscopy to calculate the TUNEL positive nuclei in epithelial cells. The TUNEL positive epithelial cells were counted in three fields of view per slide and averaged.

### Cytokine Measurements

After uterine transplantation, sera were collected at indicated time points. Peritoneal lavage fluids were collected by washing the peritoneal cavity with 400 μl PBS, then centrifuged at 20,000 g for 10 min. Supernatants were analyzed for IL-1β (DY401, R&D systems, MN), IL-1α (433403, BioLegend) and IL-33 (88–7333, eBioscience, CA) levels by ELISA kits, as per manufacturer's instructions.

### Gene Expression Analysis

Total RNA was extracted with an RNeasy Mini Kit (Qiagen, Netherlands), and cDNA synthesized using ReverTra Ace (TOYOBO, Osaka, Japan). Gene expression levels were quantified with TaqMan Gene Expression Assays (Applied Biosystems, Tokyo, Japan). Results are shown as relative expression standardized against the expression of β-actin. Specific primers and probes used for quantitative RT-PCR were *Il33* (Mm00505403_m1) and *actin-b* (4352933E) (Applied Biosystems).

### Statistical Analysis

Data are expressed as the mean ± S.D. Statistical significance was determined by the two-tailed Student's *t*-test, one-way analysis of variance (ANOVA) with Tukey's multiple range test, or two-way ANOVA with the Holm-Sidak multiple range test using Prism 6 (MDF, Tokyo, Japan). *P*-values < 0.05 were considered significant.

## Results

### Administration of IL-33 Exacerbates Endometriosis

**Figure 1:**
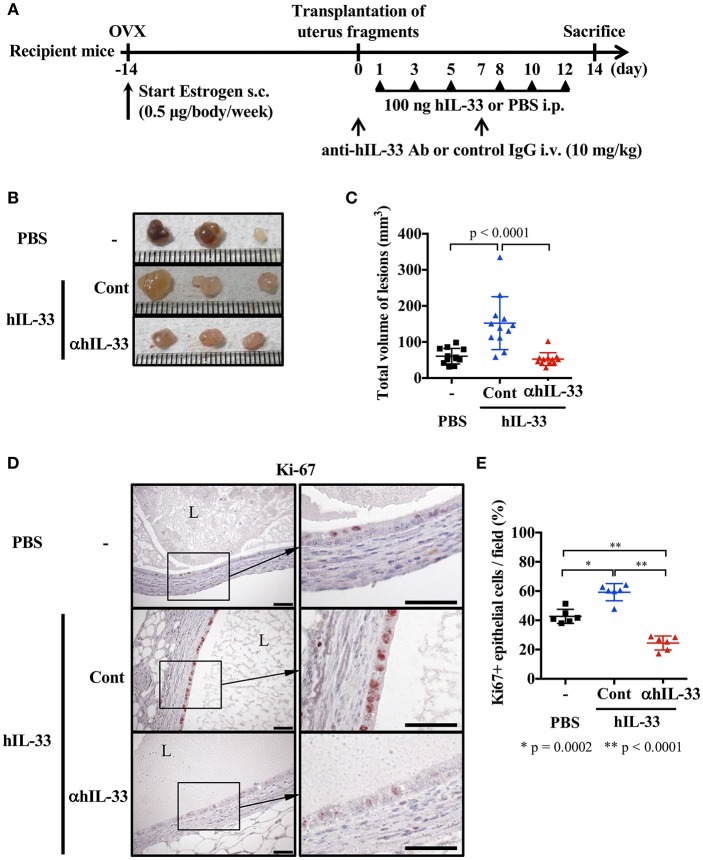
Exogenous IL-33 exacerbates endometriosis. **(A)** Experimental workflow. Wild type BALB/c mice were ovariectomized (OVX) and administered estrogen subcutaneously (s.c.) for 2 weeks before transplantation of uterine fragments. After transplantation, mice received PBS or recombinant human IL-33 intraperitoneally (i.p.) at day 1, 3, 5, 8, 10, and 12. For the neutralization experiment, IL-33-treated mice were intravenously (i.v.) injected with control IgG (Cont) or anti-hIL-33 Ab (αhIL-33) at day 0 and 7. **(B)** Representative endometriosis lesions from each mouse. **(C)** Total volume of the lesions (*n* = 12 in each group). Pooled data from two independent experiments are shown (mean ± SD). **(D)** Immunohistological staining of Ki-67; Brown. L, lumen, Scale bar: 50 μm. **(E)** The proportion of Ki-67 positive epithelial cells lining the lumen of the cyst wall (*n* = 6, mean ± SD). Statistical analyses were performed using a one-way ANOVA with Tukey's *post-hoc* tests **(C,E)**.

A previous report indicated that IL-33 levels were elevated in ascites from severe endometriosis patients (9). Therefore, we first examined the effect of IL-33 on lesions in our mouse endometriosis model. Mice were ovariectomized and treated with estrogen as described in methods, then 2 weeks later, uterine samples were transplanted into the peritoneal cavity. After transplantation, mice were administered rhIL-33 intraperitoneally three times a week and the growth of endometriosis-like lesions were examined 2 weeks after transplantation (Figure 1A). We found that the size of lesions was significantly increased by administration of rhIL-33 (Figures 1B,C). Simultaneously, Ki-67 staining of the diseased tissue revealed that rhIL-33 enhanced the proliferation of epithelial cells (Figures 1D,E). The increased lesion volume and epithelial cell proliferation induced by rhIL-33 were completely suppressed by administration of anti-human IL-33 antibody (Figures 1B–E), indicating that excessive levels of exogenous IL-33 exacerbated endometriosis in this mouse model.

### Endogenous IL-33 Is Involved in Lesion Formation of Endometriosis

**Figure 2:**
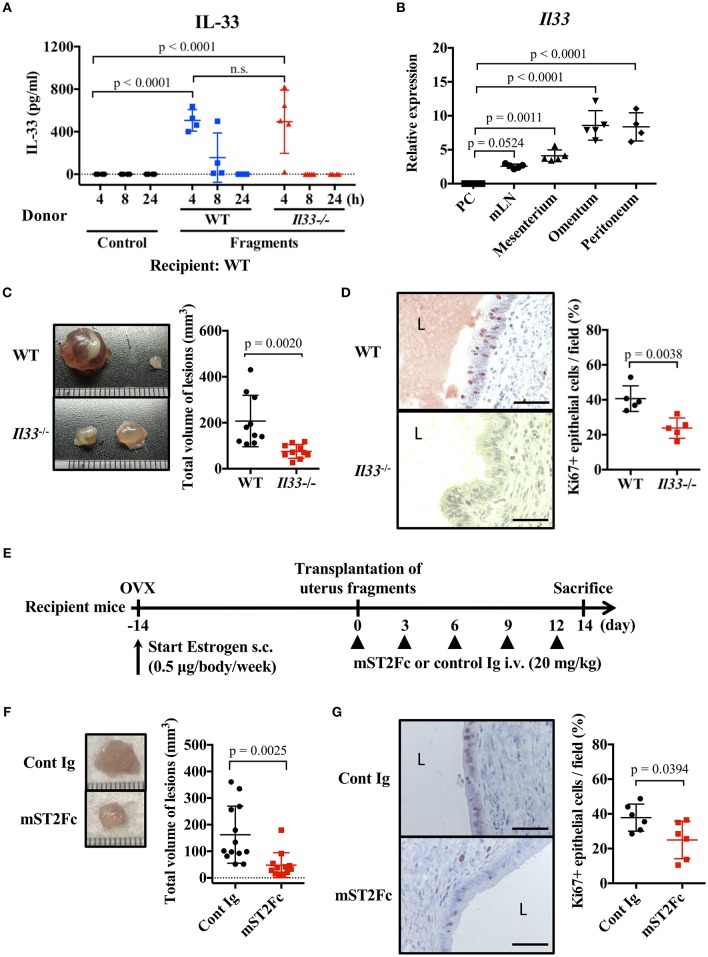
Endogenous IL-33 contributes to lesion formation in endometriosis. **(A)** Concentration of IL-33 in peritoneal lavage fluid. Wild type (WT) BALB/c mice were injected with PBS or uterine fragments transplanted from WT or *Il33*^−/−^ mice. Peritoneal lavage fluids were collected at indicated time points. **(B)** *Il33* mRNA expression in intraperitoneal tissues was measured by real-time RT-PCR. PC: peritoneal cells in peritoneal lavage fluid, mLN: mesenteric lymph node. **(C)** Mouse endometriosis model using WT mice or *Il33*^−/−^ mice. (Left) Representative endometriosis lesions from each mouse. (Right) Total volume of the lesions (*n* = 10 in each group). Pooled data from two independent experiments are shown (mean ± SD). **(D)** (Left) Immunohistological staining of Ki-67; Brown. L: lumen, Scale bar: 50 μm. Low-power image was shown in Supplementary Figure 2A. (Right) The proportion of Ki-67 positive cells in epithelium (*n* = 5, mean ± SD). **(E)** Mouse endometriosis model performed with WT BALB/c mice. After uterine fragment transplantation, mice were treated with mST2Fc or control (Cont) Ig at the indicated days. **(F)** (Left) Representative endometriosis lesion. (Right) Total volume of the lesions (cont; *n* = 13, mST2Fc; *n* = 12). Pooled data from two independent experiments are shown (mean ± SD). **(G)** (Left) Immunohistological staining of Ki-67. Scale bar: 50 μm. Low-power image was shown in Supplementary Figure 2B. (Right) The proportion of Ki-67 positive cells in epithelium (*n* = 6, mean ± SD). Statistical analyses were performed using a one-way ANOVA with Tukey's *post-hoc* tests **(A,B)** or a Student's *t*-tests **(C,D,F,G)**.

We examined the effect of endogenous IL-33 on the onset of endometriosis. First, we measured the amount of IL-33 in the peritoneal cavity after transplantation of uterine fragments. IL-33 was not detected in the control group treated with antibiotics alone after laparotomy, but was detected in the peritoneal cavity 4 h after transplantation (Figure 2A). It is known that IL-33 is released from necrotic cells, so we used *Il33*^−/−^ mice as uterine donors to confirm that IL-33 was not derived from donor cells damaged by graft fragmentation. We found that IL-33 levels were comparable when using *Il33*^−/−^ and WT donors (Figure 2A), indicating that IL-33 was produced from the recipient's cells after uterine transplantation.

To clarify the source of IL-33, we analyzed *Il33* mRNA expression in several tissues of the peritoneal cavity. We found strong *Il33* expression in the omentum and peritoneum, relatively weak expression in the mesenterium and mesenteric lymph node (mLN), and very low expression in peritoneal cells of peritoneal lavage fluid (Figure 2B). As the omentum and internal surface of the peritoneum are covered with mesothelial cells (34), uterine fragments may stimulate mesothelial cells lining the surface of the peritoneum cavity to release IL-33.

As uterine transplantation increased the concentration of IL-33 in the peritoneal cavity, we examined the influence of endogenous IL-33 on endometriosis-like lesion formation. Transplantation experiments resulted in a significant reduction in lesion size in *Il33*^−/−^ mice compared with WT mice (Figure 2C). In addition, *Il33*^−/−^ mice had a reduced proportion of Ki-67 positive epithelial cells in the lesion (Figure 2D, Supplementary Figure 2A). Furthermore, we neutralized the effects of endogenous IL-33 after transplantation by the administration of soluble mouse ST2 (mST2Fc), which contains the extracellular domain of mouse ST2 and Fc portion of human IgG (Figure 2E). We found that the lesion size and frequency of cell proliferation were suppressed by mST2Fc (Figures 2F,G, Supplementary Figure 2B). Since we observed the effect of IL-33 on the proliferation of endometrial epithelial cells, we examined the surface expression of ST2 on epithelial cells, considering that the epithelial cells may be direct target of IL-33. Unexpectedly, we could not detect ST2 expression on the surface of uterine endometrial epithelial cells (Supplementary Figures 3A,B). Taken together, these findings reveal that IL-33 was produced intraperitoneally at the onset of endometriosis, thereby promoting cell proliferation and increasing the lesion. In addition, neutralization of IL-33 suppressed the enlargement of the lesion.

### IL-1 Exacerbates Endometriotic Lesions

**Figure 3:**
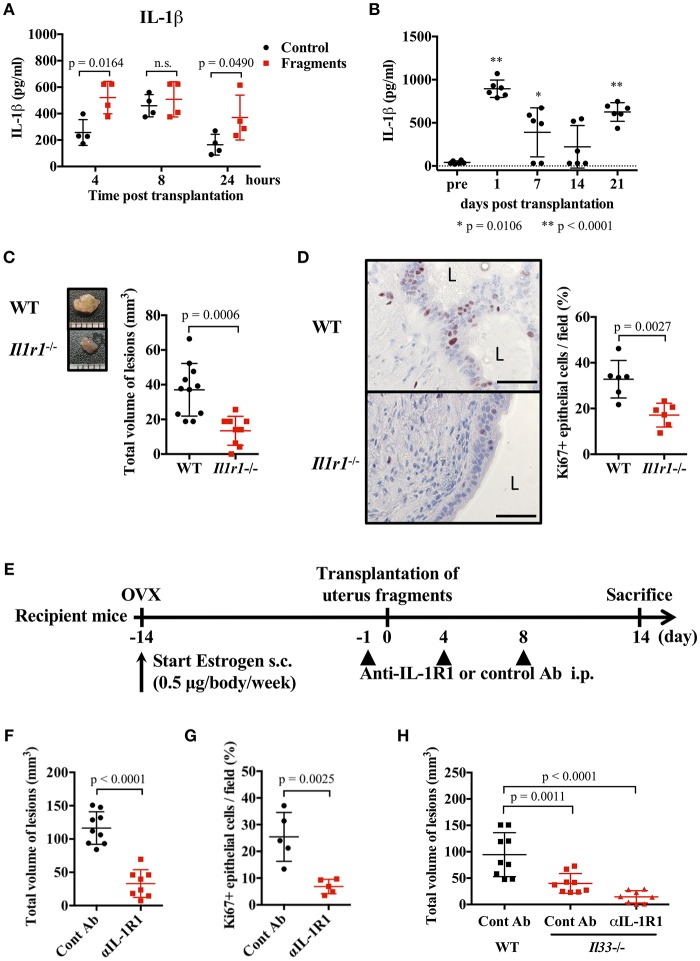
IL-1 exacerbates endometriosis lesions. **(A,B)** Concentration of IL-1β in peritoneal lavage fluids (**A**, *n* = 4), or serum (**B**, *n* = 6). Wild type (WT) BALB/c mice were injected with PBS or transplanted with uterine fragments from WT mice. Peritoneal lavage fluids or serum were collected at indicated time points. Statistical analyses were performed using a two-way ANOVA with Holm-Sidak *post-hoc* tests **(A)** or a one-way ANOVA with Tukey's *post-hoc* tests. **(C)** Mouse endometriosis model using WT C57BL/6 mice or *Il1r1*^−/−^ mice. (Left) Representative endometriosis lesion. (Right) Total volume of the lesions (WT; *n* = 11, *Il1r1*^−/−^; *n* = 9). Pooled data from two independent experiments are shown (mean ± SD). **(D)** (Left) Immunohistological staining of Ki-67. Scale bar: 50 μm. Low-power image was shown in Supplementary Figure 2C. (Right) Proportion of Ki-67 positive cells in epithelium (*n* = 6, mean ± SD). **(E)** Mouse endometriosis model performed with WT BALB/c mice. One day before and 4 and 8 days after uterine fragment transplantation, mice were treated with anti-IL-1R1 Ab (αIL-1R1) or control (Cont) Ab. **(F)** Total volume of the lesions (cont; *n* = 9, anti-IL-1R1; *n* = 8). Pooled data from two independent experiments are shown (mean ± SD). **(G)** Immunohistological staining of Ki-67 was performed. Proportion of Ki-67 positive cells in epithelium are shown (n = 5, mean ± SD). Statistical analyses were performed using a Student's *t*-tests **(C,D,F,G)**. **(H)** Mouse endometriosis model performed as in **(E)** with WT and *Il33*^−/−^ mice. Total volume of the lesions (WT: cont; n = 9, *Il33*^−/−^: cont; *n* = 9, anti-IL-1R1; *n* = 8). Pooled data from two independent experiments are shown (mean ± SD). Statistical analyses were performed using a one-way ANOVA with Tukey's *post-hoc* tests.

IL-1β is thought to act on endometrial stromal cells and to be involved in the development of endometriosis (7, 35–37). However, this proposed action of IL-1 has not been confirmed *in vivo*. Therefore, we examined the role of IL-1 in a mouse endometriosis model. First, we measured the concentration of IL-1α and IL-1β in the blood and peritoneal cavity after transplantation of uterine fragments. A transient increase of IL-1β was observed in the abdominal cavity of both the control and transplantation groups 8 h after treatment, but in the transplanted group, IL-1β was increased from 4 h and remained higher 24 h after transplantation compared with the control group (Figure 3A). IL-1α was transiently increased at 4 h (Supplementary Figure 4A). Blood IL-1β was also increased 24 h after transplantation, and gradually decreased at 2 weeks, but increased again at 3 weeks after transplantation (Figure 3B). However, no significant increase in IL-1α concentration was found in the serum (Supplementary Figure 4B).

To clarify the role of IL-1 in lesion formation, we used C57BL/6-background IL-1R1-deficient mice for the endometriosis model. As previously reported, C57BL/6 mice exhibited smaller lesion formation than BALB/c mice (38). In *Il1r1*^−/−^ mice, only very small lesions were observed compared with WT mice (Figure 3C). The number of Ki-67 positive epithelial cells was markedly decreased in *Il1r1*^−/−^ relative to WT mice (Figure 3D, Supplementary Figure 2C). To block the action of IL-1, we administrated an anti-IL-1R1 antibody after transplantation (Figure 3E), and found it strongly suppressed lesion formation and the proportion of Ki-67 positive cells (Figures 3F,G). Furthermore, when the anti-IL-1R1 antibody was administered to *Il33*^−/−^ mice to suppress both IL-33 and IL-1, lesion formation was strikingly inhibited compared with those in control mice (Figure 3H). These results reveal that IL-1 was involved in the development of endometriotic lesions.

### MyD88 Signaling Is Essential for Endometriotic Lesion Formation

**Figure 4:**
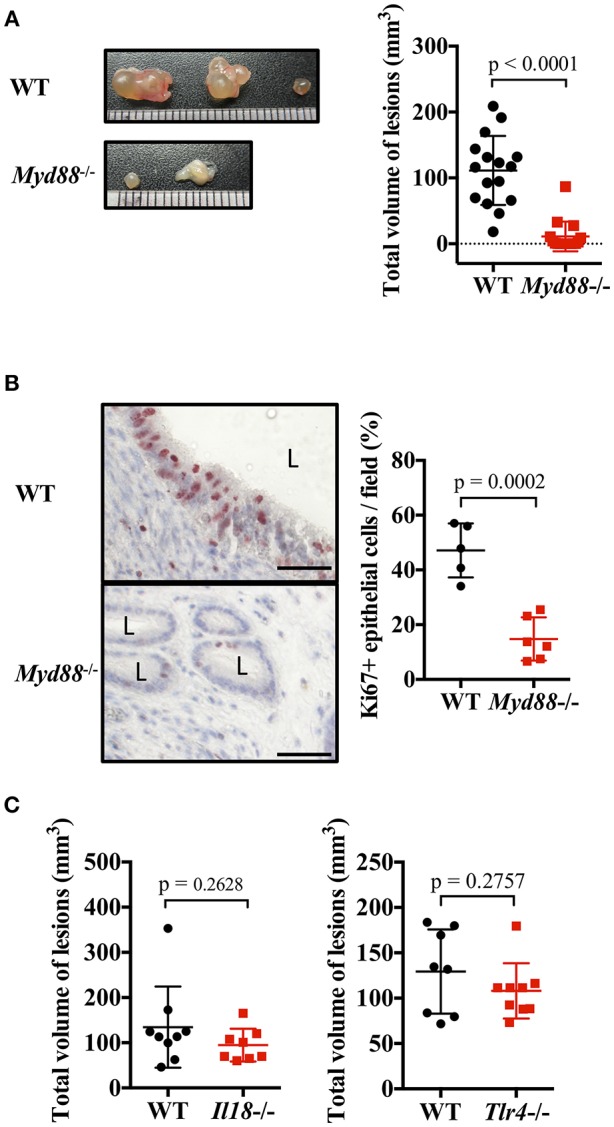
MyD88 signaling is essential for endometriotic lesion formation. **(A)** Mouse endometriosis model performed with wild type (WT) BALB/c mice or *Myd88*^−/−^ mice. (Left) Representative endometriosis lesions. (Right) Total volume of the lesions (*n* = 20). Pooled data from three independent experiments are shown (mean ± SD). **(B)** (Left) Immunohistological staining of Ki-67. Scale bar: 50 μm. Low-power image was shown in Supplementary Figure 2D. (Right) Proportion of Ki-67 positive cells in epithelium (WT; *n* = 5, *Myd88*^−/−^; *n* = 6, mean ± SD). **(C)** Mouse endometriosis model performed with WT, *Il18*^−/−^, and *Tlr4*^−/−^ BALB/c mice. Total volume of the lesions (WT vs. *Il18*^−/−^, WT: *n* = 9, *Il18*^−/−^: *n* = 8; WT vs. *Tlr4*^−/−^, WT: *n* = 8, *Tlr4*^−/−^: *n* = 9). Pooled data from two independent experiments are shown (mean ± SD). Statistical analyses were performed using a Student's *t*-tests.

IL-1 and IL-33 act via MyD88, an adapter molecule for signal transduction. Therefore, we investigated the formation of endometriosis-like lesions in MyD88-deficient mice lacking the key signaling pathway of IL-1 family cytokines. Compared with WT mice, the formation of lesions were remarkably suppressed in *Myd88*^−/−^ mice, with almost no cystic morphology observed (Figure 4A). Histological examination revealed lesions with small cystic morphology, but most epithelial cells were Ki-67 negative (Figure 4B, Supplementary Figure 2D). In addition, analysis of the frequency of apoptotic cells in endometriotic lesions found no difference between the *Myd88*^−/−^ and WT mice (Supplementary Figure 5), indicating that MyD88 contributed to the growth of epithelial cells but not to their survival. These results reveal that MyD88 is essential for cystic endometriosis lesion formation.

MyD88 is also involved in other IL-1 family cytokine and Toll-like receptor (TLR) signaling, other than IL-1 and IL-33. Therefore, the involvement of other ligands and receptors was investigated. There was no difference in the size of endometriotic lesions in IL-18-deficient mice and TLR4-deficient mice compared with WT mice (Figure 4C).

### Blockade of MyD88 Signaling Suppresses Endometriosis

**Figure 5:**
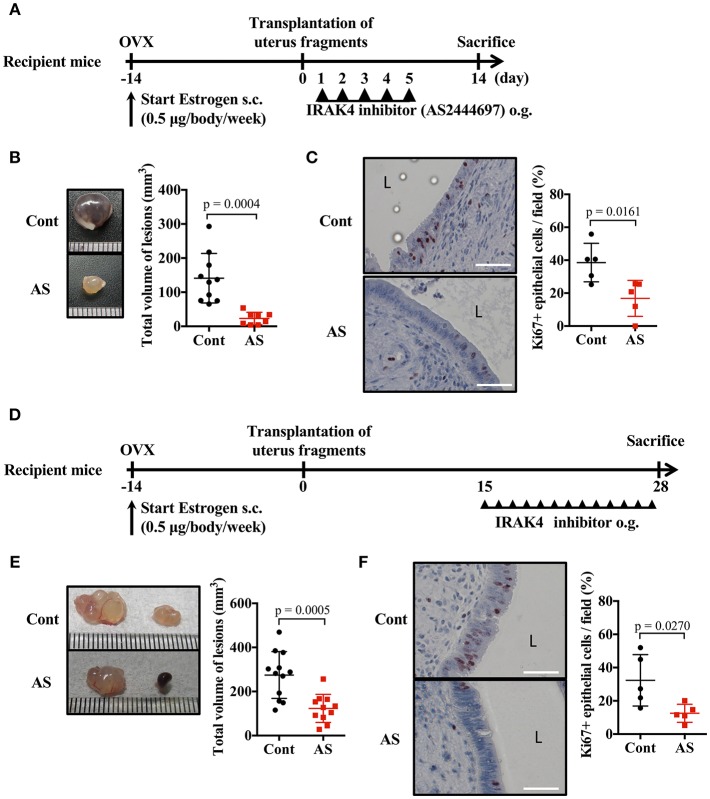
Blockade of MyD88 signaling suppresses endometriosis. **(A)** Mouse endometriosis model performed with WT BALB/c mice. After uterine fragment transplantation, mice were treated with AS2444697 (AS) or sorbent (cont) at indicated time points. o.g., oral gavage. **(B)** Total volume of the lesions (cont; *n* = 10, AS; *n* = 8). Pooled data from two independent experiments are shown (mean ± SD). **(C)** (Left) Immunohistological staining of Ki-67. Scale bar: 50 μm. Low-power image was shown in Supplementary Figure 2E. (Right) Proportion of Ki-67 positive cells in epithelium (*n* = 5, mean ± SD). **(D)** Mouse endometriosis model performed with WT BALB/c mice as in **A**. Administration of inhibitor was started at 2 weeks after transplantation. **(E)** Total volume of the lesions (cont; *n* = 12, AS; *n* = 11). Pooled data from two independent experiments are shown (mean ± SD). **(F)** (Left) Immunohistological staining of Ki-67. Scale bar: 50 μm. Low-power image was shown in Supplementary Figure 2F. (Right) Proportion of Ki-67 positive cells in epithelium (*n* = 5, mean ± SD). Statistical analyses were performed using a Student's *t*-tests.

Because the signal transduction pathway mediated by MyD88 was essential for endometriosis lesion formation, we investigated whether inhibition of IRAK4 (an essential protein kinase for this pathway) provided an effective treatment. Daily oral administration of AS2444697, an IRAK4 inhibitor, for five consecutive days after the transplantation of uterine fragments was found to inhibit epithelial cell proliferation and markedly inhibit lesion growth (Figures 5A–C, Supplementary Figure 2E).

When considering a therapeutic approach to endometriosis, it would be beneficial to be effective against formed lesions. Therefore, we administered IRAK4 inhibitor 2 weeks after uterine implantation, when lesions had already formed, and examined its effect on lesion growth (Figure 5D). Compared with the control group, the AS2444697 treated group had a significantly smaller lesion size (Figure 5E). Epithelial cell proliferation was also suppressed by AS2444697 (Figure 5F, Supplementary Figure 2F). From these results, it is clear that MyD88 signaling was involved in the early stage of lesion formation, as well as the increase of lesion size after formation. Therefore, it is possible that blocking the MyD88 signaling pathway may provide an effective treatment for endometriosis.

## Discussion

In this study, we investigated the molecular mechanism involved in endometriosis and candidate pathways as treatment targets. Using an *in vivo* murine model with transplanted uterine fragments, we revealed that cytokines using MyD88 signaling, such as IL-1 and IL-33, were induced after transplantation and were important for ectopic endometrial cell proliferation. Furthermore, we demonstrated that neutralizing antibodies to IL-33 and/or IL-1R1, or an inhibitor of IRAK4 (involved in MyD88 signaling) were effective for the treatment of endometriosis in this model.

Previous reports showed that IL-33 was present in ascites of patients with endometriosis, and IL-33 concentrations were elevated in correlation with progression of the disease state, suggesting a relationship between IL-33 and the progression of endometriosis (9). Recently, another group reported that mouse endometriosis was exacerbated by administration of IL-33 (39), consistent with our results. Using IL-33-deficient mice, we clearly demonstrated that endogenous IL-33 plays an important role in lesion formation. The role of endogenous IL-33 was further confirmed by using mST2Fc, which mimics the IL-33 neutralizing function of soluble ST2. Furthermore, by using rhIL-33 and anti-human IL-33 antibody, we demonstrated that neutralization of IL-33 was effective at inhibiting the progression of endometriosis, and the use of anti-human IL-33 antibody may provide a beneficial clinical treatment.

In the present study, the source of IL-33 was likely to be the recipient cells, because IL-33 was detected in ascites even when uterine fragment transplants were prepared from *Il33*^−/−^ mice. The expression of IL-33 in the peritoneal cavity was high in the peritoneum and omentum, which are tissues that contain a layer of mesothelial cells (34). Because IL-33 was released a short time after uterine transplantation, we propose that mesothelial cells first contact the transplanted fragments and provide the source of IL-33. Usually, IL-33 is released from the nucleus by the physical damage of tissues, but our model may not provide such a strong stimulus. We recently found that damage-associated molecular patterns (DAMPs) induce the expression of IL-33 (manuscript in preparation), and such DAMPs may be involved in the mechanism releasing IL-33, but the precise mechanism remains unknown. Here, we detected IL-33 only 4 h after transplantation, but we cannot exclude the possibility that low-level IL-33 is released continuously in the peritoneal cavity and contributes to the development of endometriosis.

Interleukin-33 uses the ST2 and IL-1RAcP as a receptor, and their expression is found in various immune system cells, such as mast cells, Th2 cells, and ILC2s (14). As ST2 was not detected on the surface of normal endometrial epithelial cells, it is possible that the immune system cells responding to IL-33 may produce secondary mediators which act on endometrial cells. Alternatively, some inflammatory conditions may induce ST2 expression on endometrial cells.

Interleukin-1β is produced by cleavage of precursor pro-IL-1β by caspase-1, which is activated via inflammasomes (40). It was reported that the NLRP3 inflammasome was involved in the exacerbation of endometriosis in a mouse model (41), consistent with our current findings showing smaller lesions in IL-1R1-deficient mice. The IL-1R1 is a common receptor for IL-1α and IL-1β. Our present work detected IL-1β in ascites and serum, and considering the involvement of inflammasomes, IL-1β may have an important role in endometriosis. However, IL-1α is also released from the nucleus when the cell is damaged (like IL-33), and we indeed detected IL-1α in the ascites at 8 hours after transplantation, thus IL-1α may also contribute to the development of endometriosis.

There are caveats for using IL-1/IL-1R1 blocking reagents to treat patients with endometriosis wishing to give birth, such as the reported IL-1R antagonist (IL-1Ra)-induced inhibition of murine embryo implantation *in vivo*, and of increased trophoblast motility by urokinase plasminogen activator and plasminogen activator inhibitors *in vitro* (42, 43). However, recent case reports demonstrated successful pregnancy during treatment with IL-1Ra in patients with familial Mediterranean fever (44) and periodic syndromes, such as adult-onset Still's disease (45). In these diseases and gout, IL-1 is an important factor causing strong pain. Indeed, recently Yu et al. reported that a potential of IL-1β in neuroangiogenesis by the induction of brain-derived neurotrophic factor from eutopic endometriosis stromal cells (8). Suppression of IL-1 may provide effective treatment against pelvic pain caused by endometriosis.

It was reported that lipopolysaccharide (LPS) exacerbates endometriosis via NF-κB activation (46). Because LPS binds to TLR4 and activates MyD88 signaling (47), the current findings using *Myd88*^−/−^ mice are consistent with LPS actions in endometriosis. Furthermore, TLR4 has been reported as a receptor for endogenous DAMPs (48). However, in our model, *Tlr4*^−/−^ mice showed normal lesion formation, so the LPS-TLR4 pathway may not be essential for lesion formation. The target cells of LPS are unknown for endometriosis, but considering LPS acts on cells such as macrophages to produce IL-1β (47), it is possible that exacerbation of endometriosis by LPS may be mediated by induction of IL-1β. In addition, it was reported that the reflux of intrauterine flora exacerbated endometriosis by bacterial contamination of menstrual blood (49).

Recently, Kaabachi et al. reported that the concentration of anti-inflammatory cytokine IL-37 was increased in the peritoneal fluid of patients with endometriosis (50). IL-37 also has a capacity to affect the occurrence and development of endometriosis in a mouse model (51). Interleukin-37 is an IL-1 family cytokine that uses the IL-18R to inhibit IL-18 signaling. Like IL-1β, IL-18 is an IL-1 family cytokine that is cleaved and activated by caspase-1. Several groups reported that IL-18 concentrations were down-regulated in peritoneal fluid of patients with endometriosis (52, 53), but other groups did not observe this decrease (54, 55). Thus, the role of IL-18 in endometriosis remains controversial. In our experiment, lack of IL-18 did not affect lesion formation in mice, similar to the lack of TLR4. However, because IL-18 is also produced from macrophages by LPS stimulation (56) and is also an activator of MyD88 signaling, it may also influence the development of disease associated with bacterial infection.

Finally, we found that an IRAK4 inhibitor suppressed the onset and growth of endometriosis. The IRAK4 protein kinase is essential for NF-κB activation downstream of MyD88, and its inhibitor blocks most signals of IL-1 family cytokines and TLRs (26, 57). This inhibitor may provide a powerful therapeutic agent capable of suppressing the augmentation of endometriosis induced by IL-1β, IL-33, and LPS. Similar inhibitory effects were obtained using anti-IL-1R1 antibodies, and a stronger inhibitory effect may be expected by combining this treatment with an anti-IL-33 antibody or soluble ST2. Thus, our findings show that IL-1 family cytokines and signaling pathways may provide valuable targets for the treatment of endometriosis.

## Data Availability

The datasets generated for this study are available on request to the corresponding author.

## Author Contributions

TK, KY, and KI carried out the experiments. KY, HS, and TY planned the experiments and supervised the study. TK and KY wrote the manuscript. KY, KM, SH, and TY analyzed the data.

### Conflict of Interest Statement

KY, HS, and TY received funding from Mitsubishi Tanabe Pharma Co. TY received materials (anti-IL-33 antibody and mST2Fc) from Mitsubishi Tanabe Pharma Co. The funder had no control over the interpretation, writing, or publication of this work. KY, TK, and TY have patent applications (PCT/JP2018/032494 and JP TOKUGAN 2018-164658) in the field of immunotherapy for endometriosis. KY had full access to all of the data in this study and KY take complete responsibility for the integrity of the data and the accuracy of the data analysis. The remaining authors declare that the research was conducted in the absence of any commercial or financial relationships that could be construed as a potential conflict of interest.

## Abbreviations

Ab: Antibody
IL-1R1: IL-1 receptor 1
IL-1Ra: IL-1R antagonist
ILC2: Group 2 innate lymphoid cell
NK: Natural killer
PBL: Peripheral blood leukocyte
Q-PCR: Quantitative PCR.
