# Supplementary figures and images for: Interleukin-1/-33 Signaling Pathways as Therapeutic Targets for Endometriosis
Source: Front Immunol. 2019 Aug 22;10:2021. doi: 10.3389/fimmu.2019.02021 (PMC6714064; doi:10.3389/fimmu.2019.02021)

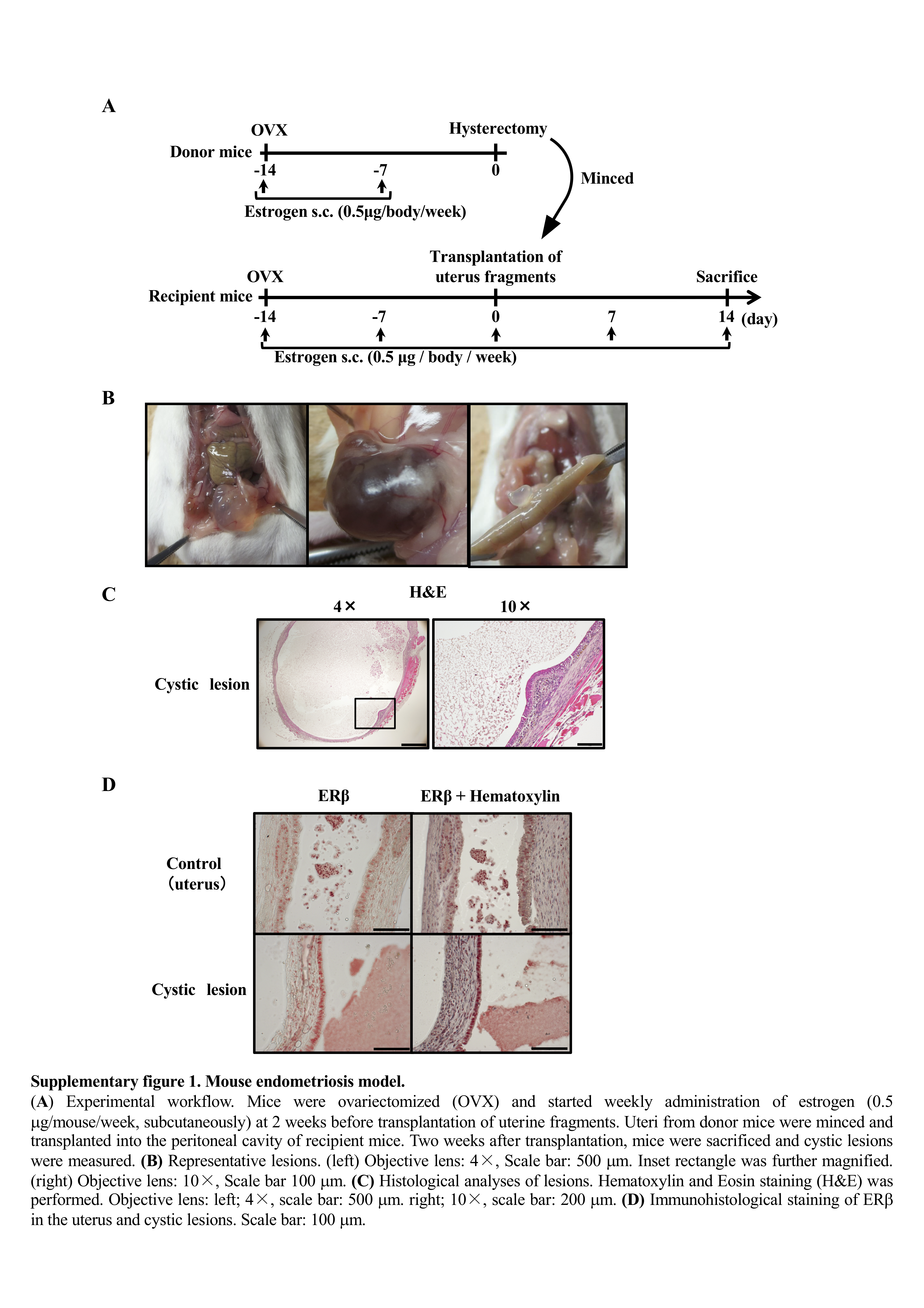

Supplement: Supplementary file 1 [file Image_1.TIFF]

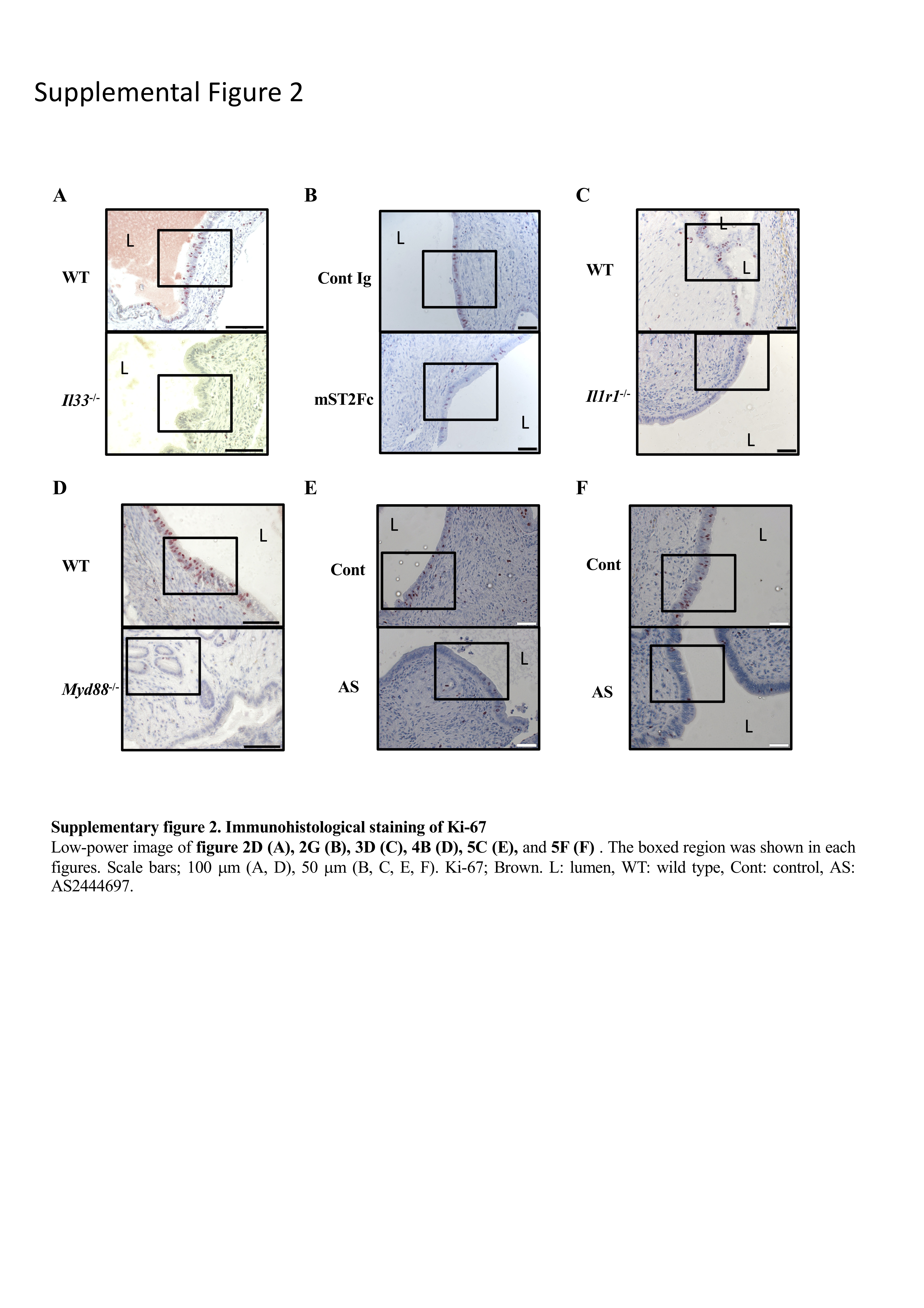

Supplement: Supplementary file 2 [file Image_2.TIFF]

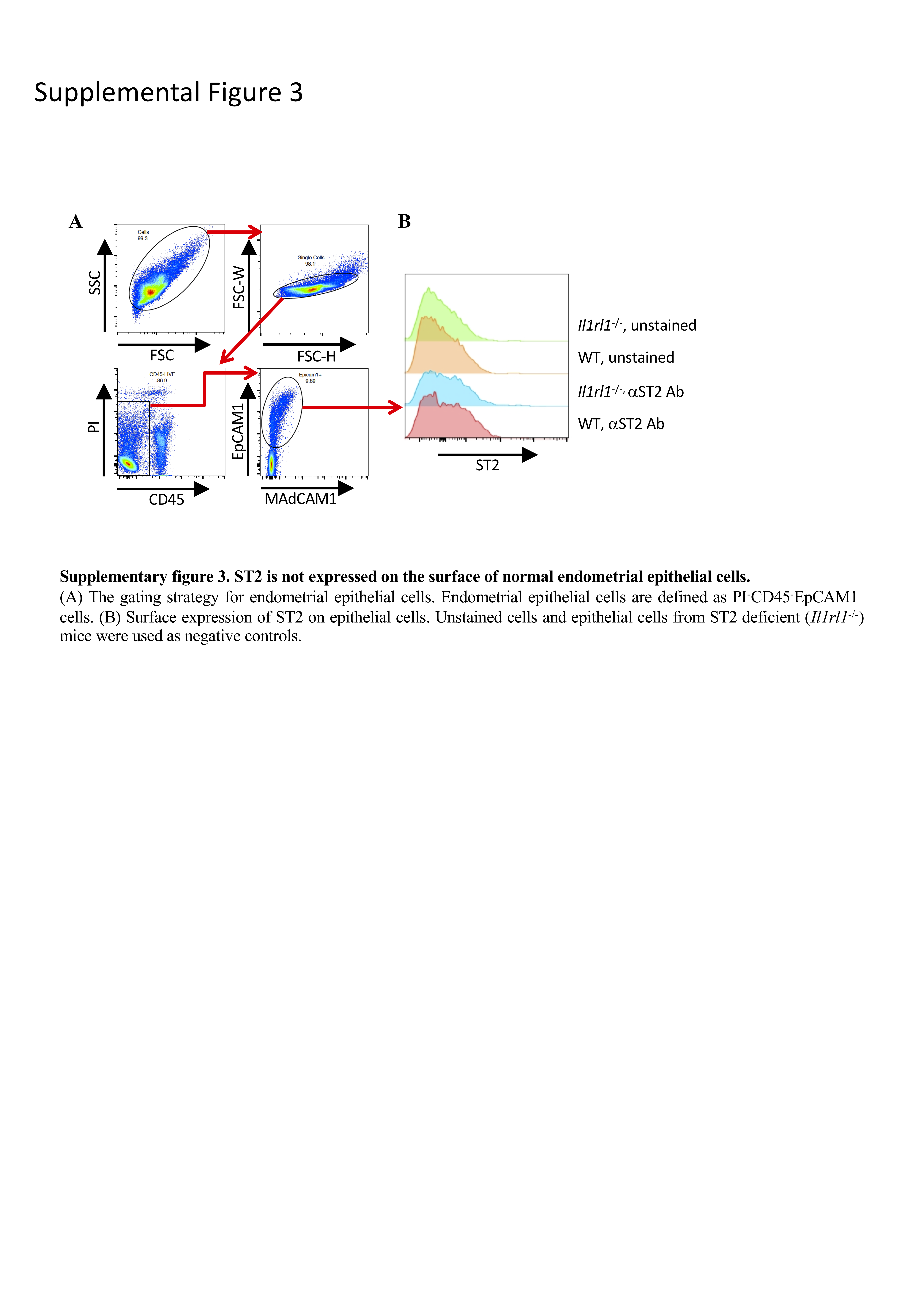

Supplement: Supplementary file 3 [file Image_3.TIFF]

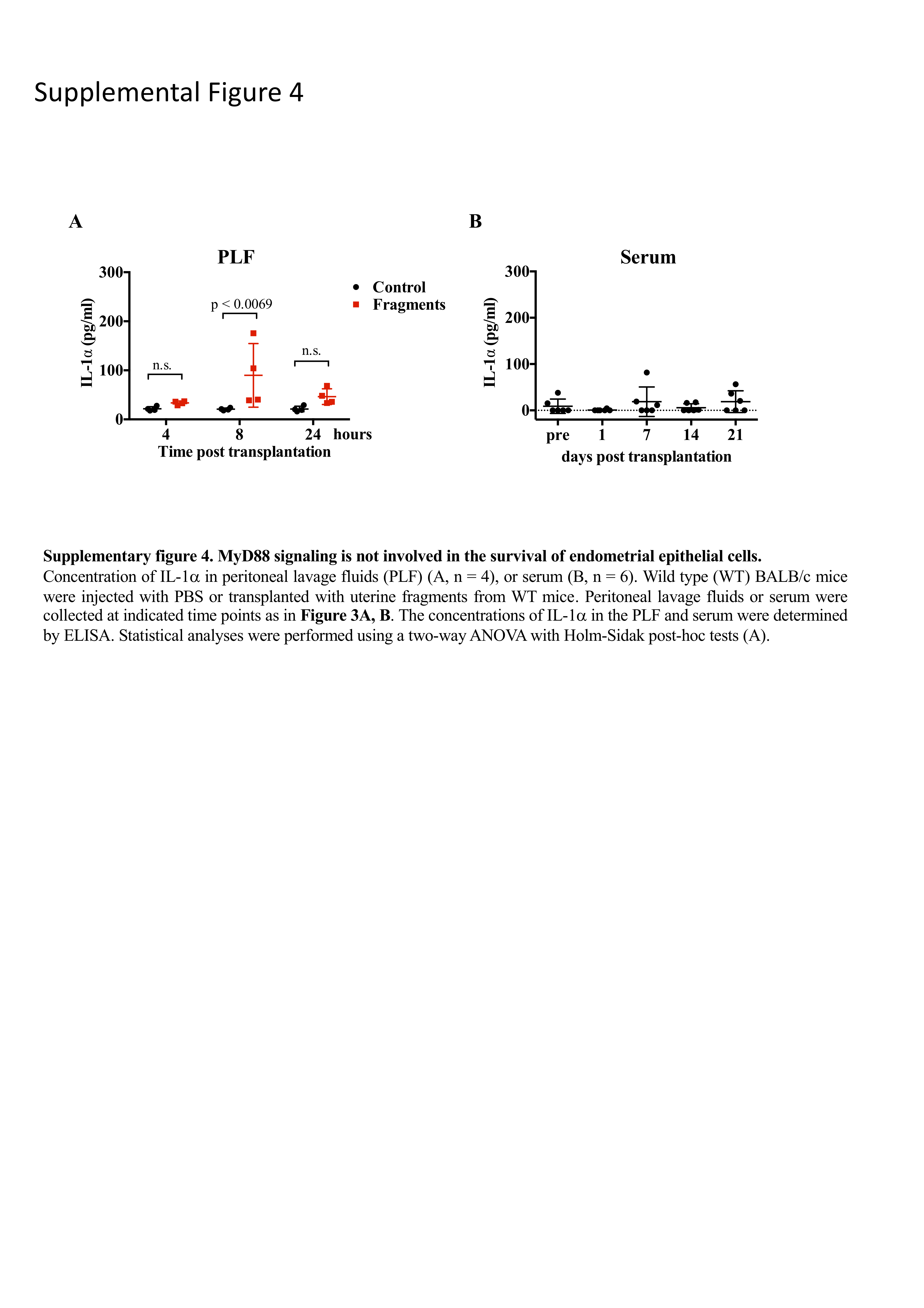

Supplement: Supplementary file 4 [file Image_4.TIFF]

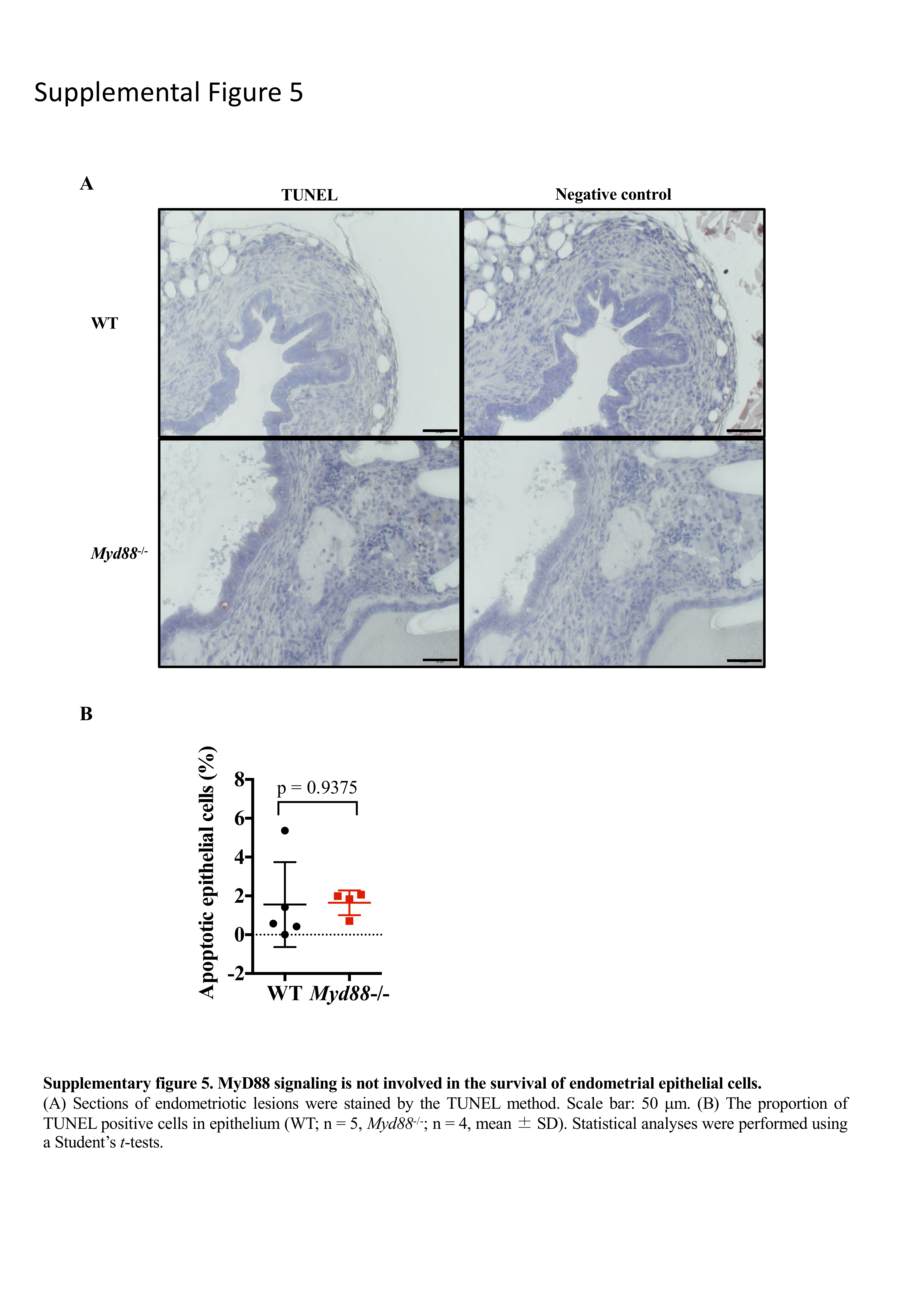

Supplement: Supplementary file 5 [file Image_5.TIFF]
